# On-Line Metrology with Conoscopic Holography: Beyond Triangulation

**DOI:** 10.3390/s90907021

**Published:** 2009-09-04

**Authors:** Ignacio Álvarez, Jose M. Enguita, María Frade, Jorge Marina, Guillermo Ojea

**Affiliations:** 1 University of Oviedo, Dept. Electrical Engineering. Campus de Viesques s/n, 33204 Gijón, Spain; E-Mails: ialvarez@isa.uniovi.es (I.A.); mfrade@isa.uniovi.es (M.F.); gojea@isa.uniovi.es (G.O.); 2 DSIPlus, Cirujeda 12, Bajo, 33205 Gijón, Spain; E-Mail: jorgemj@isa.uniovi.es

**Keywords:** optical metrology, conoscopic holography, industrial inspection

## Abstract

On-line non-contact surface inspection with high precision is still an open problem. Laser triangulation techniques are the most common solution for this kind of systems, but there exist fundamental limitations to their applicability when high precisions, long standoffs or large apertures are needed, and when there are difficult operating conditions. Other methods are, in general, not applicable in hostile environments or inadequate for on-line measurement. In this paper we review the latest research in Conoscopic Holography, an interferometric technique that has been applied successfully in this kind of applications, ranging from submicrometric roughness measurements, to long standoff sensors for surface defect detection in steel at high temperatures.

## 1. Introduction

**Figure 1.**
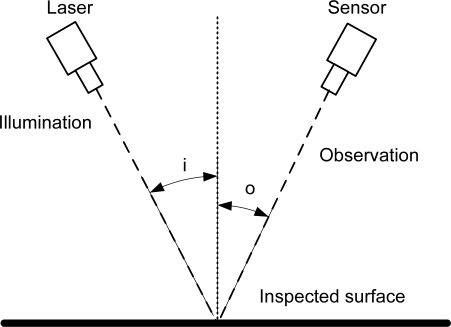
Principle of triangulation sensors.

Modern manufacturing requires on-line inspection methods to stay competitive. Metrology systems able to detect surface defects, measure dimensions and, in general, acquire data about the geometrical shape of objects in a 3-D space, are a must for quality control, industrial inspection or reverse engineering. In addition these systems must be versatile, reliable, inexpensive and easy to set up, and often must be able to work in real time to give fast feedback in the usual difficult conditions of the process line. One of the most usual methods to accomplish this task is laser triangulation. This method usually projects a small laser spot or thin line which is reflected by the object and captured by a sensor, as shown in Figure 1.

The triangulation angle between the illumination and detection branches translates depth changes in the inspected object into lateral displacements of the spot or the line in the recorded image according to Equation 1.

(1) $$\delta l=\delta z\frac{\text{sin}(i+o)}{\text{cos}(i)}$$

where *i* and *o* are the angles of incidence and observation respectively with respect to the surface’s normal, and *δl* is the lateral displacement corresponding to a depth change *δz*. By making *i* = 0 or *o* = 0 we get normal incidence or normal observation. The latter is often used, as it results in larger lateral displacements for the same depth change.

Of course, as it derives from Equation 1 and is widely known, resolution directly relates to the ability of distinguish very small shifts in the laser spot on the image, so the *density* of pixels, or ultimately, the aperture of the imaging system, is crucial.

This usually imposes a limit in the standoff of the system, as small apertures lead to short working distances, which might be an issue in harsh environmental conditions, where the sensor should be kept as far as possible from the process line. In addition, small apertures limit the range of measurement, as the laser spot quickly gets out of the image.

Resolution can be enhanced by increasing the triangulation angle, of course, but there can also exist limitations in this case, as problems with the shading effect (some parts of the object are either not illuminated or cannot be seen by the camera) and with uneven illumination increase accordingly.

**Figure 2.**
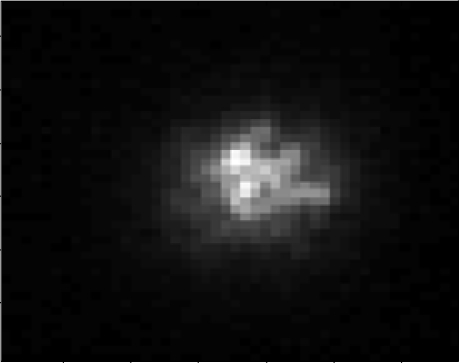
Image of a laser spot over a rough surface. The localization of the centre cannot be done without certain uncertainty, due to the speckle effect (courtesy of SPIE).

Whichever being the final setup of the triangulation system, there are two fundamental physical effects that impose a limitation to the final precision. The first one is speckle noise, which has been examined in depth by Häusler [1]. Speckle is an interference effect caused by the microtopology of the inspected surface, due to the spatial coherence of the illuminating source. The result is a pattern of dark and light spots superimposed to the image, which adds uncertainty to the localization of the laser spot, as shown in Figure 2.

This noise cannot be reduced by simply averaging over several acquisitions, as the speckle pattern will remain constant due to the spatial coherence. For averaging to be useful, the acquisitions should be made from different points of view or over different places over the surface. This second option occurs naturally when the inspected object is in movement, although there is a loss of lateral (and probably depth also) resolution due to the same averaging process that is removing speckle. We will focus on speckle later on, in Section 4.

The second physical effect that has to be taken into account is the smallest displacement of the point in the image that can be measured. Even using sub-pixel detection methods, there is a physical limit that cannot be overcome: the Rayleigh resolution limit.

Resolving the position of two light sources (or even the detection of the presence of both) is possible only if they are separated at least by a distance *δl* given by Equation 2.

(2) $$\delta l=1.22\frac{f\;\lambda }{D}$$

where λ is the wavelength of the light source, *f* is the focal length of the detector’s lens system, and *D* the diameter of the objective lens’ aperture.

The reasoning behind this phenomenon is diffraction, and the Rayleigh criterion defines the smallest shift that can be detected in a triangulation system, which, in turn, defines the maximum depth resolution of the system, simply by inserting Equation 1 into Equation 2. This criterion may *slightly* underestimate the resolution in some specific cases, but real inspection systems are usually far from reaching it.

So a discouraging deadlock exists, as the improvement of depth resolution can only be obtained by increasing the triangulation angle or decreasing the working distance, which both might be an issue in a real system.

This physical limitation can be improved using interferometric techniques, as will be discussed in the next section.

## 2. Beyond the Rayleigh Limit with Conoscopic Holography

Interferometric techniques make use of the interference pattern generated when two coherent sources arrive at the detection plane with a small optical path difference; this difference generates in a detector an image composed of dark and white fringes, from which the distance information can be extracted.

Usually this is done by splitting the laser light and making it travel through different paths, with one set as the reference and the other being reflected by the inspected object. In this way, it is possible to recover shape information from an object by making both beams interfere.

These setups have problems when applied in on-line inspection systems, as they normally need very careful setups to avoid misalignments and are quite sensitive to environmental conditions such as vibrations or air turbulence. This lack of robustness mainly derives from the fact that both wavefronts travel through different paths. Fortunately there are some techniques that do not have this limitation, the so-called *common-path* interferometric techniques. Conoscopic Holography is one of them.

### 2.1. Conoscopic Holography

**Figure 3.**
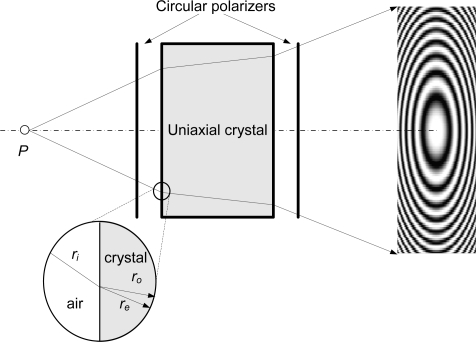
Conoscopic Holography working principle (courtesy of SPIE).

Conoscopic Holography is an incoherent-light interferometric technique, based on the double refraction property of uniaxial crystals which was developed by Sirat and Psaltis in 1985 [2]. The basic system is shown in Figure 3.

When a polarized monochromatic light ray crosses an uniaxial crystal, it is divided into two orthogonal polarizations, (ordinary and extraordinary rays) which travel at different speeds through the crystal. The speed of the ordinary ray is constant; however, the speed of the extraordinary ray depends on the angle of incidence. In order to make both rays interfere in the detector plane, two circular polarizers are placed before and after the crystal. This basic system is called *conoscope* and the interference pattern obtained in the detector is a *Gabor zone lens*, defined by the equation:

(3) $$I=I_{0}\left(1+\gamma_{0}\;\text{cos}\left(K\frac{r^{2}}{Z_{c}^{2}}\right)\right)$$

where *K* depends on the opto-geometrical parameters of the system and the wavelength, *γ*_0_ is known as fringe visibility, *r* is the radial distance from the center of the *Gabor zone lens*, and *Z_c_* is the so-called conoscopic corrected distance, the geometrical mean distance of the ordinary and extraordinary rays to the recording plane. The difference between the conoscopic corrected distance and the real distance is negligible here, as they are both nearly equal but for a constant additive factor.

The resulting pattern has a radial symmetry, so all the information is contained in one radius. Therefore, it is possible to calculate the original distance to the light emitting point from the fundamental frequency of one of the lines of the signal, with the appropriate calibration. Punctual conoscopic devices use a linescan CCD to acquire this signal.

### 2.2. Linear Conoscopic Holography

A further improvement is the linear Conoscopic Holography [3, 4], where two crystals, with a specific orientation one with respect to the other, build up the conoscopic module. In this configuration, the wavefront reflected by the inspected surface is duplicated by the conoscope, and the resulting wavefronts emerge with a lateral shear between them. The obtained interference pattern consists of equidistant linear fringes with frequency depending almost linearly on distance following the expression:

(4) $$I=I_{0}\left(1+\gamma_{0}\;\text{cos}\left(2\pi \frac{K^{\prime }}{Z}\right)\right)$$

where *K′* depends mainly on the source wavelength and the thickness of the crystal, and *Z* is the distance between the specimen and the detection plane.

This technique does not need spatial coherence in the light source. It only needs the light to remain quasi-monochromatic for the interference pattern to remain stable during the acquisition of one frame. Usually laser light is used for convenience, which means speckle is present. But if somehow the speckle effect can be reduced (measuring in movement or dealing with specular surfaces, for example), the resolution of the system is well below the Rayleigh limit.

Finally, another good advantage is that the illumination branch can be set up collinearly with the detection branch. This way the system can look into holes and sharp edges, and there are no shading effects, while still attaining very high precisions and providing very fast measurements.

A similar system, using a Savart Plate instead of a conoscope, was developed by Häusler et al[5] back in 1988, and the authors make a good report of its main characteristics and limitations. Most of their conclusions are also applicable in this case.

### 2.3. Examples

Sensors based on this principle are manufactured by Optimet Optical Metrology Ltd., owners of the patent for Conoscopic Holography. They report sensors able to perform point measurements at 3000 Hz, with precisions in the order of the micron for standoffs of 18.5 mm and below 12 *μ*m for standoffs of 90 mm.

**Figure 4.**
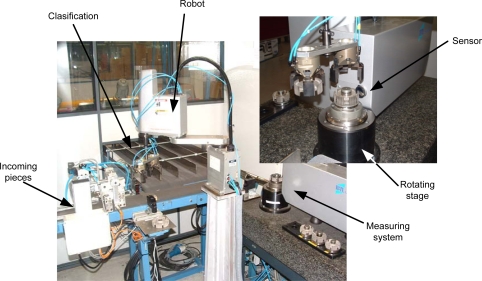
On-line measuring system and classification of synchronizer hubs, installed at PMG Asturias.

At DSIPlus and the University of Oviedo, several on-line inspection systems based on this technology [6, 7] have been developed. Figure 4 shows an example of an on-line system for quality control of sintered automobile synchronizer hubs. A pick&place robot takes each hub from the incoming line and places it onto a rotation stage. An optical measuring sensor based on Conoscopic Holography (Conoprobe) acquires the 3-D data which is processed by a computer. Every piece is inspected, measured and classified automatically, according to its main diameters, that are measured with nearly *±*1 *μ*m precision. All this operation is done without interfering with the production process and without adding any delays, with a cycle time (load-measure-unload-classify) of less than 5 seconds. Data is stored for later analysis and reference.

**Figure 5.**
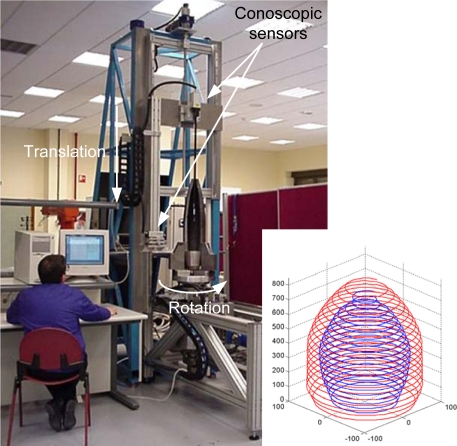
System for measuring the internal and external surfaces in a solid of revolution simultaneously, taking benefit of the collinear setup of Conoscopic point sensors.

Another interesting example is shown in Figure 5, where the collinear setup of the Conoprobe shows its benefits. In this system the internal and external surfaces of projectiles were measured simultaneously. The projectile is rotated while the sensors are moved downwards to acquire the 3-D data. The internal surface is measured by introducing a one meter length periscope that bends the light path by 90°.

Other applications of these sensors include the measurement of low frequency vibrations in hostile environments [6], where traditional methods (accelerometers) can not be used (high temperature target) or give inaccurate results (very low frequencies).

## 3. Including Triangulation

Collinear setups such as those described above offer many advantages, and are optically designed to generate a signal with large frequency differences along the measurement range. This provides the high sensitivity and accuracy of these devices, but needs to use short focal length lenses and short standoffs for high accuracy in small ranges. For many industrial applications this is not a valid solution, as they require large standoffs due to the process or plant conditions. In many occasions these industrial applications do not need to obtain absolute measurement values given by frequency processing, but it is enough to obtain relative measurements or displacements: this is the case for surface defects detection and surface characterization.

To achieve better resolutions from large standoffs when only relative measurements are needed, the University of Oviedo in close collaboration with Optimet, worked in a modification in the setup which consists in introducing triangulation between the laser source and the detection branch, so variations in the distance do not only provide (very small) fringe frequency variations but also the whole interference pattern is shifted. The equation now becomes:

(5) $$I=I_{0}\left(1+\gamma_{0}\;\text{cos}\left(2\pi \frac{K^{\prime }}{Z}+\varphi \right)\right)$$

With this setup, the phase of the signal *φ* is also related to the distance of the emitting point. In fact, by using the phase information, resolution is improved by more than 10 times [8].

In this case the main drawback is that it is limited to the range [0*,* 2*π*), so values differing 2*π* cannot be distinguished. This limits the actual uses of the sensor to obtain only *relative* measurements of objects with smooth surfaces, where the condition of continuity can be applied to resolve the 2*π* ambiguities (known as *phase unwrapping* [9, 10]). We will focus on this limitation in Section 5.

The small triangulation angle needed (over 5 degrees) still enables to produce a compact robust sensor, and the shading effects are negligible if we apply the constraint of smooth objects. For most applications, the system can be considered quasi-collinear.

### 3.1. Long-standoff Profilometer

**Figure 6.**
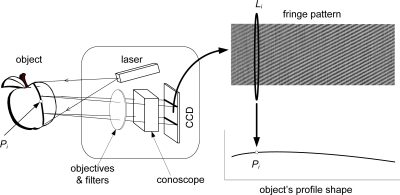
Linear sensor including triangulation (courtesy of SPIE).

It is possible to project a thin laser line instead of a single point onto the inspected object and, by means of an adequate optic system including a cylindrical lens, each point resulting of the intersection from the laser plane with the object generates a one dimensional signal related to its distance, which is recorded by a conventional CCD camera (see Figure 6).

For each point *P_i_* of the intersection between the laser line and the object, a one-dimensional cosine signal is recorded on the CCD (*L_i_*). By processing this signal the distance to the original point can be obtained. Processing all the column lines in the fringe pattern results in a measure of the profile distances. This way it is possible to obtain a height profile in a single frame.

Results for this large standoff profilometer in industrial applications have been reported in [11–14] among others. An outline of one of them follows.

### 3.2. Example: On-line Surface Inspection of Hot Steel Slabs

One of the steps in the production of steel sheets is the continuous casting, where the liquid steel is poured in a bottomless mould to obtain a continuous solid steel bar. This bar is then cut into slabs of the appropriate dimensions, with a length of 7–12 m, a width of 700–1,600 mm and a weight around 20 t. During the solidification in the casting process, under some temperature and manufacturing conditions, thermal tensions may produce cracks on the surface of the slab. In these cases, the slab should be repaired before sending it to the rolling stage, which actually means three days of cooling for manual inspection, reparation if needed, and finally reheating it again to continue with the process.

The basic objective of the inspection system (described in depth in [11]) is to detect these surface defects automatically and without affecting the throughput of the plant. The working conditions are very adverse, with high levels of electromagnetic noise, dust, vibrations, and hot materials.

**Figure 7.**
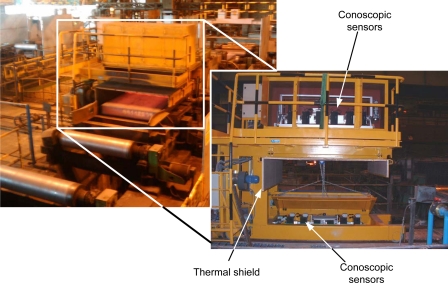
External view of the inspection system installed in Arcelor (Spain).

The temperature of the slab at this point is between 650 and 900 °C, so sensors should be kept at a safe distance; in this case it has been chosen to be 1,200 mm (see Figure 7). Also, the high temperature generates air turbulence that is an important source of noise for non common-path devices.

**Figure 8.**
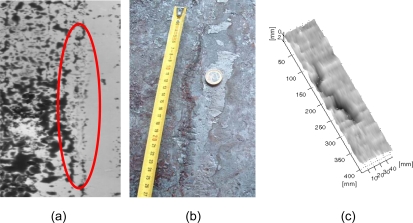
A typical surface crack; (a) shows a gray-level image of the hot slab, where the crack (marked by the ellipse) is hardly visible, (b) shows a picture of the crack once the slab is cold, and (c) shows the distance map obtained with the inspection system over the hot slab. Undulations along the surface are most probably due to vibrations in the slab while it is moved by the rollers (courtesy of SPIE).

Typical cracks have a longitudinal shape with a length that varies from 100 mm up to more than 1 meter, a width over 1 mm, and a depth that is usually more than 0.5 mm. Figure 8 shows a typical crack over the surface of a slab and the correspondent distance map. Cracks are much clearer once the slab is cold, as it is in the solidification process when they open completely.

The system has been working in a Continuous Casting plant since January 2007 with high reliability and productivity: more than 7 million tons of steel have been inspected so far.

## 4. Reaching Sub-Micron Precisions: Attacking Speckle

In Section 1, the two basic limitations of triangulation techniques were introduced. We have dealt with the Rayleigh limit, but precision is also limited by speckle noise, if coherent light is used. This noise also affects interferometric techniques, adding uncertainty to the calculation of the central frequency of the fringes, and therefore, the phase.

Several approaches can be used for reducing the influence of the speckle noise. A moving target enables the different speckle patterns to be integrated in each acquisition, reducing the global speckle noise; this reduction technique is naturally used in many industrial inspection applications and improves depth resolution over a 50%, and it is what was used in all the above examples. For further noise reduction, two-dimensional filtering methods based on the directionality of the fringes have been developed and tested in several conditions, and are reported in [15]; their drawback is the loss of resolution both in the depth and in the lateral axes.

When using Conoscopic Holography, however, it is possible to reduce the coherence of the illuminating source as long as it remains quasi-monochromatic, well focused and with enough power to obtain fringe patterns with good contrast. This way speckle noise would be minimized, increasing depth resolution in the apparatus.

### 4.1. Speckle Reduction Methods

Many authors have investigated speckle reduction techniques in different fields. One of the most common methods consists in placing a diffuser after the light source, with a small lateral displacement, and making it rotate[16]. This produces a series of different speckle patterns over the illuminated surface that are averaged in time during the acquisition of a frame. Speckle contrast is reduced when the number of different patterns due to the rotation of the diffuser is high enough when compared with the camera frame rate. However, the light source becomes partially diffusive and there are problems when trying to obtain a small spot or a collimated beam. More advanced methods are discussed in [16–19], for example.

For the initial sensor prototype described in [20], a rotating diffuser was used, as it is the simplest method, but other alternatives are currently under study. The diffuser is placed after the laser unit (685 nm, 50 mW) with a small lateral displacement and slightly out of focus. A small DC motor makes it rotate at a controllable speed. The light coming out of the diffuser is captured by a condenser and passed through a small window. Then it is transformed into a line and projected onto the inspected sample. First experiments were made with a 120 grit ground-glass diffuser, but it seems that better results are obtained with holographic diffusers of small diffusion angle (0.5°).

With such a system it is possible to attain sub-micron precision from standoffs as large as 100–160 mm and triangulation angles in the order of 25°. The sensor is able to obtain profiles of 10–12 mm in length in a single acquisition, with a lateral resolution of 10 *μ*m. The depth of field is in the order of 4 mm, which is very large for this type of devices. The equivalent triangulation sensors have a depth of field below one millimeter.

The Root-Mean-Square (rms) error along the profile for several consecutive measures is below 0.08 *μ*m for those working distances, and mostly caused by instability in the signal (due to the rotating diffuser) and camera noise.

### 4.2. Example of Application: Roughness Measurements

In order to study the capabilities and behavior of the system, a prototype was built using a 1.5 mm thick linear conoscopic module, a 685 nm/50 mW laser and a working distance of 110 mm. Four galvanized steel specimens were scanned with this system. Areas of 12 *×* 0.5 mm were analyzed and compared to the data obtained with a stylus-type profilometer. Because it is very difficult to scan *exactly* the same profile, direct comparison is not possible. Thus, data were compared by using roughness parameters such as Roughness Average (*R_a_*) or Root-Mean-Square Roughness (*R_q_*). The results shown in Table 1 are very promising, and the deviations from the measurements done with the stylus are below the *±*10% level, which is expected between two different mechanical instruments.

However, another limitation arises strongly when high precisions are intended. As the phase values are limited to the range [0*,* 2*π*) the presence of large steeps in the object’s surface may result in ambiguous measurements. In the next section, our current research for dealing with this problem is shown.

## 5. Extending the Measurement Range: Multiple Wavelength Conoscopic Holography

The use of the phase information reduces the maximum steep that can be measured without ambiguity. The fact that the phase values are limited to the interval [0, 2*π*) (or [*−π, π*)) limits the maximum steep that is measurable without ambiguity to half the distance at which the phase signal wraps. With current setups, which provide high resolutions, this means that steeps as small as one micron may be incorrectly measured, which, in turn, might become unacceptable for many applications.

A possible technique to overcome this limitation is based on the use of more than one wavelength [21–24], so that the unambiguous region is extended to half the equivalent wavelength, which can be calculated as:

(6) $$\lambda_{\mathrm{eq}}=\frac{\lambda_{1}\lambda_{2}}{\left|\lambda_{2}-\lambda_{1}\right|}$$

Thus, the use of closer wavelengths provides higher range and more insensitivity to chromatic aberrations, but also they are also more sensitive to noise, which is also scaled by a factor inversely proportional to the wavelength difference. However, there have been some attempts to minimize this effect (i.e., see [21]). Although well-known from some time, there is still a lot of research and development in the area of two and multiple-wavelength interferometry [25, 26].

### 5.1. Multiple Wavelength Conoscopic Profilometry

Our approach combines two laser sources of different wavelengths in a beam combiner and passes the resulting beam through the rotating holographic diffuser. In the resulting interference pattern the so-called *beat phenomenon* (a low frequency envelope typical when two sinusoidal signals are combined) is observed. Two peaks, one associated to each wavelength, appear in the frequency domain, making it possible to extract both phase signals which can be combined to increase the maximum slope measurable without ambiguity.

The experimental prototype sensor consists of two lasers of 685 nm and 660 nm with powers of 50 mW and 100 mW respectively, with external power control. In this way it is possible to adjust the power of the individual lasers so that two clear peaks about the same amplitude appear in the frequency spectra. Also, it is simple to adapt the power according to the reflective properties of the surface, and to use just one of the wavelengths if necessary.

The wavelengths are chosen so that they are close enough to provide an important improvement in the measurement range and reduce errors due to chromatic aberrations, while still being correctly separated when performing signal processing. Both the illumination and the acquisition branches work at a distance of 100 mm from the test specimen, which was mounted on a precision stage with lateral movement for 3D scanning.

**Figure 9.**
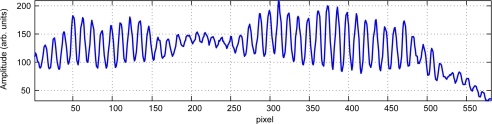
Obtained signal corresponding to a single column. A low-frequency beat phenomenon is observed (courtesy of SPIE).

**Figure 10.**
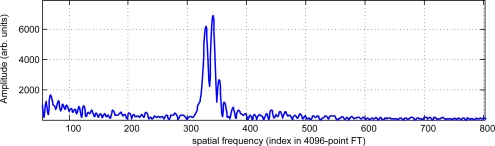
Detail of the 4096-point Fourier Transform of the signal in Figure 9. Two peaks at indexes 331 and 343 appear clearly (courtesy of SPIE).

The obtained fringe pattern for a single column shows the beat formation seen in Figure 9, where one of the columns of a fringe pattern is plotted. The frequency spectrum of this line (Figure 10) clearly shows two peaks with maxima at indexes 343 and 331. The ratio between those maxima (1.0363) strongly agrees with the nominal ratio between the laser wavelengths of 685 nm and 660 nm (1.0379).

Once the two individual phase fronts *φ*_1_(*x*) and *φ*_2_(*x*) have been extracted, the phase at the synthetic wavelength *φ*(*x*) can be obtained by simple substraction:

(7) $$\varphi (x)=\varphi_{1}(x)-\varphi_{2}(x)$$

Repeatability is quite high along the whole profile. Measuring 15 consecutive acquisitions shows an average standard deviation of *σ* = 0.0384 radians for the combined phase.

By using close wavelengths and getting their data set simultaneously, some common sources of error outlined by Cheng *et al.* [21] are reduced. Air turbulence cannot affect the wavefronts differently, as they are taken at the same time, so these errors are also reduced [27]. In addition, there is no data matching problem. The chromatic aberrations introduced by the system affect similarly at both wavefronts, so they are almost cancelled.

With this setup, steeps of approximately 3 *μ*m are correctly resolved, while still retaining precisions well below the micron. The behavior of a prototype of this sensor has been reported in [28, 29].

### 5.2. An example: Beating the Limits of Triangulation

A simple experiment can demonstrate how this technique can beat the limits of laser triangulation. The Rayleigh limit for our prototype sensor can be calculated with Equations 1 and 2, knowing that $D=\frac{f}{f/\#}$, as:

(8) $$\delta z=1.22\;\lambda f/\#\frac{\text{cos}(i)}{\text{sin}(i+o)}$$

We use an objective lens with *f* = 75 mm, *f/*# = 1.8, λ = 660 nm (a second laser with λ = 685 nm is used for applying multiple-wavelength techniques, but only one would be used in laser triangulation), and our triangulation angle is about 25° in a specular reflection setup (*i* = *o ≈* 12.5°), so Equation 8 gives a result of *δz* = 3.47 *μ*m. Therefore, height variations in the profile below this limit cannot be distinguished by triangulation methods, even without speckle noise.

**Figure 11.**
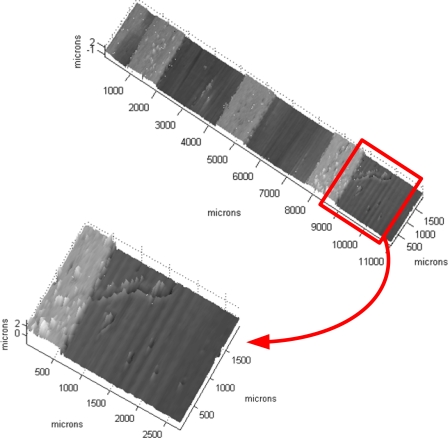
Detail of the 3-D surface scan of the test specimen. In addition to the one micron steps, other smaller details, such small scratches and defects, are clearly seen (courtesy of SPIE).

**Figure 12.**
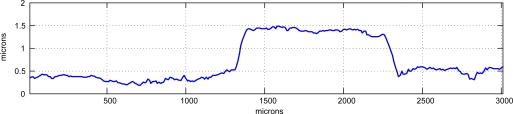
Detail of a profile obtained for the test specimen.

Using interferometry it is possible to obtain precisions beyond this limit. A test specimen made of bands of resin of one micron in height (approximately) over a silicon wafer substrate was scanned with our prototype. The resulting 3-D map is shown in Figure 11 and a detail of one profile in Figure 12, where these bands are clearly seen as well as other smaller details, showing the precision of the system is below the micron scale.

## 6. Conclusions and Future Trends

Common-path interferometric techniques such as Conoscopic Holography are ideal for on-line industrial applications due to their simplicity, high stability, and immunity against vibrations and harsh environmental conditions. Our experience with this technology shows that it is a good candidate for optical metrology in this kind of applications, when high precisions that cannot be obtained with classical triangulation techniques are required.

The working principle and the main lines of research have been outlined, showing how they do improve its range of applications. These novelties include a small triangulation setup, performing measurements using the phase information to increase the precision when large standoffs are needed, and speckle reduction techniques to obtain profile measurements with precisions well below the micron from safe standoffs. Finally a work in progress towards extending the measurement range by using multiple-wavelength techniques has been introduced.

Intended applications include on-line roughness analysis and surface micro-defect detection, but the same principles can be applied in macro-systems, as long as the setup is scaled accordingly to the needs.

There is still a lot of room for improvement: optimizing the optical setup, searching for better, more stable, speckle reduction techniques, using different illumination (such as blue lasers, which exhibit less speckle, or superluminiscent LEDs), and using more than two wavelengths for larger ranges and more robust measurements. Nevertheless, this kind of systems is promising in dealing with some of the challenges that on-line industrial inspection pose to the metrology science at present and in the future.

## Figures and Tables

**Table 1.** Comparison between *R_a_* and *R_q_* factors in microns calculated using our optical device and the data obtained from an stylus (courtesy of SPIE).

Specimen	Optical (*μ*m)		Stylus (*μ*m)	
	*R_a_*	*R_q_*	*R_a_*	*R_q_*
A	0.312	0.400	0.307	0.409
B	0.618	0.780	0.674	0.825
C	0.300	0.374	0.286	0.393
D	0.675	0.841	0.737	0.922
